# A survey of extended-spectrum beta-lactamase-producing *Enterobacteriaceae* in urban wetlands in southwestern Nigeria as a step towards generating prevalence maps of antimicrobial resistance

**DOI:** 10.1371/journal.pone.0229451

**Published:** 2020-03-04

**Authors:** Olawale Olufemi Adelowo, Odion Osebhahiemen Ikhimiukor, Camila Knecht, John Vollmers, Mudit Bhatia, Anne-Kirstin Kaster, Jochen A. Müller

**Affiliations:** 1 Department of Environmental Biotechnology, Helmholtz Centre for Environmental Research—UFZ, Leipzig, Germany; 2 Environmental Microbiology and Biotechnology Laboratory, Department of Microbiology, University of Ibadan, Ibadan, Nigeria; 3 Otto-von-Guericke-Universität Magdeburg—Institute of Apparatus and Environmental Technology, Magdeburg, Germany; 4 Institute for Biological Interfaces (IBG5), Karlsruhe Institute of Technology, Eggenstein-Leopoldshafen, Germany; Panstwowy Instytut Weterynaryjny - Panstwowy Instytut Badawczy w Pulawach, POLAND

## Abstract

In many countries, emission of insufficiently treated wastewater into water bodies appears to be an important factor in spreading clinically relevant antimicrobial resistant bacteria. In this study, we looked for the presence of *Enterobacteriaceae* strains with resistance to 3^rd^ generation cephalosporin antibiotics in four urban wetlands in southwestern Nigeria by isolation, whole genome sequencing and qPCR enumeration of marker genes. Genome analysis of multi-drug resistant and potentially pathogenic *Escherichia coli* isolates (members of the widely distributed ST10 complex) revealed the presence of the extended spectrum beta-lactamase gene *bla*_CTX-M-15_ on self-transmissible IncF plasmids. The gene was also present together with a *bla*_TEM-1B_ gene on self-transmissible IncH plasmids in multi-drug resistant *Enterobacter cloacae* isolates. A *Citrobacter freundii* isolate carried *bla*_TEM-1B_ on an IncR-type plasmid without discernable conjugation apparatus. All strains were isolated from a wetland for which previous qPCR enumeration of marker genes, in particular the ratio of *intI1* to 16S rRNA gene copy numbers, had indicated a strong anthropogenic impact. Consistent with the isolation origin, qPCR analysis in this study showed that the *bla*_CTX-M_ gene was present at an abundance of 1x10^-4^ relative to bacterial 16S rRNA gene copy numbers. The results indicate that contamination of these urban aquatic ecosystems with clinically relevant antibiotic resistant bacteria is substantial in some areas. Measures should therefore be put in place to mitigate the propagation of clinically relevant antimicrobial resistance within the Nigerian aquatic ecosystems.

## Introduction

Antimicrobial resistance (AMR) is a global problem that threatens progress in health and the achievement of sustainable development goals [1]. Although resistance to antimicrobials is an ancient phenomenon which evolved without human influence in the past, the current increased global presence of AMR is driven by anthropogenic activities [2]. The introduction of antibiotic resistant bacteria and genes (ARB and ARG) into the environment *via* waste streams from anthropogenic sources is being increasingly recognized as an important contributing factor for its prevalence in the human population [3,4]. There are indications that in Low and Lower-Middle Income Countries (LIC and LMIC) insufficient sanitation infrastructure and the attendant release of untreated or poorly treated wastewater into the environment contributes more to the prevalence of AMR than antibiotic consumption itself [5]. Consequently, polluted aquatic ecosystems have been recognized as important reservoirs of AMR in recent times and deserve further attention [6].

Next to lacking sufficient sanitation infrastructure, most LIC and LMIC have not yet adopted national AMR surveillance programs. Such programs constitute a basis for implementing best patient-treatment practices, identifying newly emerging threats, and directing actions and resources for the containment of AMR to where they have the highest impact. Although several LMIC are taking steps towards establishing surveillance programs, their implementation is lagging, largely due to insufficient numbers of AMR diagnostic laboratories [7]. However, establishing and operating more laboratories in LIC and LMIC will need substantial economic resources, trained personnel and time. Until these are put in place, monitoring AMR profiles in selected water bodies with anthropogenic impact could be a cost-effective proxy for a national surveillance program [8]. The rational selection of environmental sampling sites is of critical importance for the success of this strategy.

The overall aim of our work is to design and implement approaches for effectively gathering knowledge on AMR prevalence in anthropogenically impacted ecosystems in LIC and LMIC that could be translated into public health-related practices. To this end we have been assessing the level of environmental contamination with clinically relevant ARG in Nigeria [9–11], the most populous nation in Africa and a LIMC. Results from these studies provided information about the genetic context of such ARG in bacteria isolated from Nigerian aquatic ecosystems. The level of AMR contamination in four polluted wetlands in Lagos and Ibadan appeared to be moderate to high, based on the relative abundance of three AMR indicator genes (*intl1*, *sul1*, *sul2*[4]) versus copy numbers of the bacterial 16S rRNA gene [12].

In this study, we investigated the same urban wetlands for the occurrence of members of the *Enterobacteriaceae* that are resistant to 3^rd^ generation cephalosporins (3GC) *via* production of extended spectrum beta-lactamases (ESBL) or AmpC beta-lactamases. The presence of such microorganisms in the environment is of high concern since they can cause difficult-to-treat infections which will place a high morbidity burden on the society [13]. ESBL genes in *Enterobacteriaceae* are frequently located on self-transmissible plasmids together with various other ARGs [14–16], while the *ampC* gene can be located on the chromosome or on a plasmid and can confer resistance to 3GC when overexpressed due to mutations [13,17]. Most common ESBL fall into one of three groups named CTX-M, TEM, and SHV, of which the CTX-M enzymes are the most prevalent [14,18,19]. The linkage of *bla*_CTX-M_ with plasmids may be a key contributor to its epidemiology in the human population, for which there are no distinct boundaries between clinical settings and community [15]. Here, 3GC-resistant *Enterobacteriaceae* were isolated from a wetland in Ibadan with high anthropogenic impact. The prevalence of these microbes correlated with qPCR-based enumeration of the relative abundance of *intI1* copy numbers, indicating that measuring this abundance alone could be sufficient for assessing the distribution of AMR in a watershed.

## Materials and methods

### Isolation of 3^rd^ Generation Cephalosporin-Resistant Bacteria from wetland sediments

Bacteria showing resistance to 3^rd^ generation cephalosporins (3GC-RB) were isolated from the sediments of four polluted wetlands in Lagos [Abule Agege (06.5145°N, 03.4002°E), Ogbe Creek (06.5135°N, 03.3937°E)] and Ibadan [Awba (07.4468°N, 03.8763°E), Apete (07.4577°N, 03.8828°E)] in southwestern Nigeria. The wetlands receive untreated wastewater and raw sewage from student hostel facilities of two universities (Awba, Apete and Abule Agege), waste streams from fish farms (Awba and Abule Agege), a Zoological Garden (Awba), and seepages from upland solid waste dumpsites (Apete and Ogbe Creek) (see [10] for a map). Detailed knowledge on quantity and quality of the various water sources in the regions is not available. Sediment samples were collected monthly between October 2014 and January 2015 and processed for the isolation of bacteria as described previously [10] except that the agar plates were supplemented with ceftriaxone (4 μg/ml). Briefly, the samples were serially diluted in saline and 3GC-RB isolated from the samples by selective enrichment on ceftriaxone-supplemented Eosine Methylene Blue agar plates. Morphologically distinct colonies selected from the agar plates were purified by re-streaking on fresh ceftriaxone-supplemented agar plates. All isolates were stored in glycerol broth (15% v/v) at -20°C for further processing. Phenotypic detection of ESBL production was carried out by the double disc synergy test (DDST) as described by the Clinical and Laboratory Standards Institute [20].

Total genomic DNA extracted from DDST-positive 3GC-RB (DNeasy Blood and Tissue Kit, Qiagen) were used as templates in PCR with primers 27F and 1492R [21] targeting the 16S rRNA gene for bacterial identification. PCR products were purified (QIAquick PCR Purification Kit, Qiagen) and sequenced with primer 27F (GATC, Konstanz, Germany).

### Minimum Inhibitory Concentration (MIC) determination

MIC (minimum inhibitory concentration) test strips containing ceftazidime (CAZ), aztreonam (ATM) (both with concentration range of 0.016–256 μg/ml), imipenem (IMP), ciprofloxacin (CIP) (both with concentration range of 0.002–32 μg/ml), and sulfamethoxazole (SMX) (0.064–1024 μg/ml) (Liofilchem Diagnostici, Italy) were used in the determination of the MIC on Müller-Hinton agar plates according to the manufacturer’s instructions. Agar plates were inoculated with standardized saline suspension (0.5 McFarland Standard) of each isolate prepared from overnight cultures. Test strips of each antibiotic were carefully layered on the inoculated plates which were then incubated at 35°C for 18 hrs. Results were interpreted with the Clinical and Laboratory Standards Institute (CLSI) MIC interpretive criteria for *Enterobacteriaceae* [20].

### Whole genome sequencing of ESBL-producing *Enterobacteriaceae*

Whole genome sequencing (WGS) was carried out as previously reported [10]. In total, 12 members of the *Enterobacteriaceae* (7 *Escherichia coli*, 4 *Enterobacter cloacae* and 1 *Citrobacter freundii* isolate) were selected for WGS. Genomic DNA (500–1000 ng) of each isolate was sheared with a Covaris S220 sonication device (Covaris Inc.; Massachusetts, USA) with the following settings: 55 seconds, 175 W, 5% Duty factor, 200 cycles of burst, 55.5 μL input volume. Sequencing libraries were prepared using the NEBNext® Ultra^™^ DNA Library Prep Kit for Illumina® (New England Biolabs, Frankfurt, Germany) following manufacturer's instructions and the libraries were sequenced with an Illumina® MiSeq machine using v3 chemistry and paired-end approaches with 301 cycles per read. Raw sequences were subjected to adapter clipping and quality trimming using Trimmomatic [22], and processed reads were assembled with SPAdes v3.6.2 [23]. Assembly quality and taxonomic placement of the genomes were assessed with CheckM v1.0.4 [24] and automated annotations were performed with Prokka v1.11 [25]. Multi locus sequence typing (MLST), Single Nucleotide Polymorphism (SNP) analysis as well as the detection of antibiotic resistance gene markers and virulence genes and plasmid incompatibility groups were performed using MLST 1.8 [26], CSI Phylogeny 1.4 [27], ResFinder 2.1 [28], VirulenceFinder 1.5 [29] and PlasmidFinder 1.3 [30], respectively, available from the Center for Genomic Epidemiology (http://genomicepidemiology.org). Prophages were searched for using PHASTER [31] and by manual inspection.

### Reference-based *in silico* plasmid assembly

Plasmid draft assemblies were generated by using reference plasmids as previously described [32]. Here, contigs of apparent plasmids and of uncertain replicon origin were sorted from those of putative chromosomal origin via MAUVE alignments between the reference strains (GenBank accession numbers in parenthesis) *E*. *coli* K12 substr. MG1655 (NC_000913), *E*. *cloacae* subsp. *cloacae* ATCC 13047 (CP001918), *C*. *freundii* CFNH1 (NZ_CP007557) and the concatenated contigs of the new isolates [33]. Annotations of the genes on potential plasmid contigs were manually inspected and contigs containing at least one *rep*, *tra* or *trb* gene were used as query sequences for BLASTn searches in the GenBank. Full plasmid sequences harboring close homologs to the aforementioned genes were downloaded from GenBank and used in MAUVE alignments with the contigs of potential plasmid origin from the novel strains. Reference plasmids belonged to the incompatibility groups IncF, IncH and IncR, respectively, and are listed in S1 Table. Furthermore, the Illumina reads of the novel strains were mapped against the plasmid references using default parameters on Geneious Prime 2019 (https://www.geneious.com). These mapped reads were re-mapped against the contigs of apparent chromosomal origin of the strains isolated in this study in order to check that no potential plasmid sequences had been overlooked. Then, initial draft assemblies were generated from all contigs of putative plasmid and of uncertain replicon origin. The drafts were iteratively refined by a combination of contig ordering and omission of some contigs based on MAUVE alignments with the reference plasmids, Illumina read mapping, and *in silico* replicating insertion sequences present in multiple copies on the respective plasmid.

### Conjugation assay

The presence of plasmids in the novel isolates and their self-transmissibility were tested *via* conjugation assays with *Aeromonas aquatica* AE235 (DSMZ 100827) as recipient strain. The donor isolates and *A*. *aquatica* were cultured in LB broth and incubated at 30°C on a shaker (120 rpm). When donor and recipient cell counts reached 10^5^ cells/ml and 10^6^ cells/ml, respectively, 100 μl from each culture were mixed in a 1.5 ml Eppendorf tube and incubated at 37°C for 1 hr. The cell suspension was vortexed and then plated on Aeromonas Isolation Agar plates with ceftriaxone (50 μg/ml) but without ampicillin typically applied to this medium. The type strain *A*. *aquatica* AE235 did not grow with ceftriaxone concentrations of 12.5 μg/ml and above. The plates were incubated at 30°C overnight after which *A*. *aquatica* colonies were counted to calculate transformation efficiencies. Three randomly selected colonies were then re-streaked and incubated at 30°C overnight on Aeromonas Isolation Agar plates with 50 μg/ml ceftriaxone to obtain presumptive *A*. *aquatica* transconjugants.

The presence of the respective plasmids in the presumptive transconjugants were confirmed by PCR-based detection of plasmid backbone genes (targeting *traA*, *traN*, *traU*, *trbB*) and ARG-containing contigs [targeting *bla*_*CTX-M-15*_, *aph(6)-Id*, *aph(3")-Ib*, *aadA5*, *aac(6')-Ib-cr*, *aac3-IIa*, *tet(B)*]. Primers were designed using the NCBI primer blast tool (https://www.ncbi.nlm.nih.gov/tools/primer-blast/) (S2 Table). DNA was extracted by the microwave boiling method [34] from randomly selected single colonies of transconjugants grown overnight. The following reaction mixture was used for PCR amplification: 1 μl extracted DNA, 0.50 μl primers (10 μM, 0.25μl each), 6.25 μl Red Taq 2 X Mastermix diluted to 12.5 μl with ddH_2_O. Initial denaturation at 95°C for 5 min was followed by 30 cycles of amplification (denaturation at 95°C for 30 sec, annealing for 30 sec at the primer pair-specific temperature shown in S2 Table, and extension at 72°C for 30 sec) and ending with final extension at 72°C for 5 min. DNA isolated from the donor strains and *A*. *aquatica* AE235 were used as positive and negative controls, respectively.

### PCR screening for beta lactamase genes in non-sequenced isolates

The presence of class A (Ambler classification scheme) ESBL genes (*bla*_CTX-M_, *bla*_GES_, *bla*_PER_, *bla*_SHV_, *bla*_TEM_, *bla*_VEB_), class B *ampC* beta-lactamases (*bla*_CMY-1,_ *bla*_CMY-2,_ *bla*_ACC,_ *bla*_ACT,_ *bla*_DHA_ and *bla*_FOX_), and class D oxacillinase genes (*bla*_OXA-1,_ *bla*_OXA-2,_ *bla*_OXA-10,_ *bla*_OXA-23,_ *bla*_OXA-24,_ *bla*_OXA-48,_ *bla*_OXA-51,_ *bla*_OXA-58_) was screened by PCR as previously described [35,36]. PCR primers and annealing temperatures are provided in S2 Table. *Klebsiella pneumoniae* strain Mnasey (*bla*_CTX-M-15_, *bla*_SHV*-*11_, *bla*_TEM-1B_), *Acinetobacter baumannii* AYE (*bla*_VEB_), *A*. *baumannii* 308 (*bla*_*OXA*-58_), *A*. *baumannii* ABIsac_coliR (*bla*_*OXA*-23_), *A*. *baumannii* OXA24 (*bla*_*OXA*-24_), *E*. *coli* CMUL64 (*bla*_*OXA*-48_) and *Providencia rettgeri* H1736 (*bla*_*OXA*-10_) were used as positive controls.

### Quantitative PCR analysis

Total community DNA samples previously extracted from the wetland sediments [12] were pre-screened to query for the presence of beta-lactamase genes *bla*_CTX-M_, *bla*_TEM_, *bla*_*SHV*_, and *ampC* using primers and conditions reported before [37,38]. Based on the results of the PCR screening, the relative abundances of Group 1 *bla*_CTX-M_ [18] and *ampC* genes in the total sediment community DNA of the four wetlands were determined by SYBR Green-based real-time PCR with four technical replicates per sample. The assay was run on a StepOne Plus Cycler (Applied Biosystems) in a 20 μl reaction volume. The primers and conditions for qPCR assays were described before for Group 1 *bla*_CTX-M_ [37] and *ampC* [38]. The abundance of *intI1* and 16S rRNA genes in the samples were determined in our previous study [12] using published primers [39,40]. Here, standards were PCR-amplified fragments of the *bla*_CTX-M_ and *ampC* genes from environmental isolates obtained in this study. Abundance of total *E*. *coli* was carried out by measuring the *uidA* gene by qPCR with the primers and conditions reported previously [41]. DNA concentrations of the standards were measured by Nanodrop spectrophotometry and the copy number (CN) per gram of wetland sediment was calculated using the relation:

$$CN=(c\;x\;6.022\;x\;10^{23})/660\times N$$

where *c* is the measured DNA concentration (μg/μl) and N is the DNA fragment length in bp.

Efficiency values were 89.9% for *bla*_CTX-M,_ 89.3% for *ampC*, and 95% for *uidA*.

## Ethics statement

There are no specific permits required for sample collection in the field studies. The wetlands are not protected and not privately owned, hence there are no regulations that restrict collection of sediment samples for research purposes from the four wetlands. The field study does not involve any endangered or protected species, only sediment samples were taken from the sites.

## Results and discussion

### Third Generation Cephalosporin-Resistant Bacteria were isolated from all investigated wetlands

A total of 90 colonies representing all different morphotypes (based on size, colour, surface texture and colony edge) growing on the ceftriaxone-supplemented agar plates from all wetland sediment samples were subjected to DDST to confirm ESBL production (41 colonies from Awba, 28 from Apete, 17 from AbuleAgege and 4 from Ogbe Creek). Thirty-five of the isolated 3GC-RB tested positive for ESBL production and were identified by partial 16S rRNA sequencing as belonging to the *Enterobacteriaceae* genera *Escherichia* (7 strains), *Enterobacter* (4 strains), and *Citrobacter* (1 strain), and to the “*Pseudomonas putida* group” [42,43] (10 strains; all with >99% identity to *P*. *monteilii*, *P*. *taiwanensis*, and *P*. *plecoglossicida*), *Caulobacter* (9 strains; about 95% identity with *C*. *segnis*), *Achromobacter* (2 strains; 98.61% identity with *A*. *insuavis*, and 99.12% identity with *A*. *spanius*, respectively), and *Stenotrophomonas* (2 strains; >99% similarity with *S*. *pavanii*). ESBL-producing bacteria have been isolated from aquatic ecosystems in different parts of the world [44–49] including Nigeria [9,50], in particular from sites were insufficiently treated wastewater is released into the aquatic environment. In the present study, ESBL production was more frequent among isolates from Awba (*n* = 23) compared to Apete (*n* = 5), Abule Agege (*n* = 7) and Ogbe Creek (*n* = 1). All ESBL-producing *Enterobacteriaceae* were from Awba wetland located within the campus of the University of Ibadan. In Abule Agege and Ogbe Creek all ESBL producers were identified as *Caulobacter* sp., while *Caulobacter* sp. and *Pseudomonas* spp. were identified as ESBL producers in Apete. This isolation pattern of 3GC-RB might reflect the different anthropogenic impact on the four wetlands. Enumerations of *intI1*, *sul1*, and *sul2* by qPCR in a previous study indicated that Awba and Abule Agege were more impacted with AMR contamination than Apete and Ogbe Creek[12].

### Overview of the draft genomes of the ESBL-producing *Enterobacteriaceae*

In order to analyze the genetic basis of 3GC resistance in the isolated *Enterobacteriaceae* their genomes were sequenced. The remaining DDST-positive isolates were not further investigated since they were outside the target phylogenetic group and PCR screening did not detect any of the tested beta-lactamase genes. Full-length 16S rRNA gene sequences showed that the sequenced strains affiliated with *E*. *coli* (n = 7), *E*. *cloacae* (n = 4) and *C*. *freundii* (n = 1) with draft genome assemblies of 4.8 Mb, 5.1 Mb, and 5.2 Mb in size, respectively. Further genome assembly characteristics are provided in Table 1.

**Table 1:** Genome and assembly characteristics of sequenced ESBL-producing *Enterobacteriaceae* from Awba wetland in Ibadan, Nigeria.

Species	*Escherichia coli*							*Enterobacter cloacae*				*Citrobacterfreundii*
Strain	CC8	CC33	CC43	CC46	CC57	CC78	CC85	CC14	CC80	CC81	CC90	CC12
**Contigcount** **[No]**	132	116	102	116	128	147	131	49	50	54	52	62
**Total length [bp]**	4829442	4834548	4835215	4835939	4833152	4839817	4831178	5115359	5114627	5114450	5115533	5224871
**Largestcontig length [bp]**	322872	486845	486949	322531	322866	322874	322858	1333559	1333328	864367	1039204	496047
**N50[bp]**	119697	135953	136131	126103	119689	125865	125819	322567	281165	280608	322434	256736
**L50 [No]**	13	11	11	13	13	12	12	4	5	6	5	8
**N80 [bp]**	42717	46326	50186	45118	44350	44358	43402	144212	124130	124217	123229	124335
**L80 [No]**	34	29	28	32	32	33	33	11	12	14	12	17
**Theoretical coverage [x]**	164	116	122	137	152	127	162	32	29	120	67	32

N50 = smallest contig of the size-sorted contigs that make up at least 50% of the respective assembly

L50 = number of contigs that make up at least 50% of the respective total assembly length

N80 = smallest contig of the size-ordered contigs that make up at least 80% of the respective assembly

L80 = number of contigs that make up at least 80% of the respective total assembly length

The genomes of all *E*. *coli* isolates (strain designations: CC8, CC33, CC43, CC46, CC57, CC78, CC85) were almost identical. Gene content was the same (disregarding the hypothetical duplication of some transposases at contig ends) and SNP counts ranged from 19 to 42. Likewise, the genomes of the *E*. *cloacae* isolates (strain designations: CC14, CC80, CC81, CC90) were almost identical with SNP counts ranging from 12 to 57.

Results from the PlasmidFinder pipeline indicated that the isolated *E*. *coli* strains carried an IncF plasmid (replicon type II-IA-IB). The conjugative plasmids of the IncF family range in size from 45 to 200 kb [16,51,52]. They seem to have a narrow host range specific to the *Enterobacteriaceae*, being mostly found in *E*. *coli*. Furthermore, they are the predominant plasmids involved in the dissemination of *bla*_CTX-M-15_ and are widely reported in bacteria isolated from humans, animals and the environment [52]. IncFII-IA-IB plasmids carrying *bla*_CTX-M_ have been implicated in community-associated infections on several continents, emphasizing the epidemic nature and global distribution of these plasmids [53,54]. The *E*. *cloacae* genomes contained contigs with similarities to IncH-like plasmids of replicon types IncH12 and H12A. These are low copy number plasmids with a wide host range, varying in size from 75 to 400 kb. They have been found in various Gram-negative microbes, and frequently carry ESBL genes alongside various other ARG [16]. The *C*. *freundii* genome contained sequences with similarities to IncR plasmids, which range in size from 40 to 340 kb and are less frequently detected although they appear to have broad host range [16]. Detailed information on these plasmid types in the novel isolates is provided in the next section.

The *E*. *coli* and *E*. *cloacae* strains were assigned by MLST to sequence type (ST) 617 and ST976, respectively, and *C*. *freundii*CC12 was assigned to ST323. *E*. *coli* ST617 is a member of the ST10 complex, which has emerged as important carrier mediating the spread of antimicrobial resistance genes including *bla*_CTX-M-15_ [55]. Members of the ST10 complex have a broad host range [56] and have been isolated from patients around the world including Nigeria [57,58], animals [59–61], food [62], and other environmental sources [48,49,63]. We are not aware of any published information on *E*. *cloacae* ST976 and *C*. *freundii* ST323 other than their mentioning in EnteroBase [64].

Several virulence genes (*ast*, *capU*, *gadAB*, *iss*, and genes for the biosynthesis and uptake of the siderophore aerobactin) were found in the genomes of the sequenced *E*. *coli* strains, which suggest that the isolates could be pathogenic. The *astA* gene codes for an aggregative heat-stable enterotoxin [65]; *capU* is predicted to code for a hexosyltransferase in enteroaggregative *E*. *coli* [66]; *gadAB* code for a glutamate decarboxylase which is involved in acid resistance in habitats such as the stomach [67]; the *iss* gene product promotes immune evasion by increasing serum survival [68]; and aerobactin is a virulence factor during urinary tract infections [69]. No potential virulence gene was detected in *E*. *cloacae* CC14, CC80, CC81, CC90 and *C*. *freundii* CC12.

All isolates were multi-drug resistant based on their ARG content, and several identical resistance genes were present in members of the three species (Table 2). The *E*. *coli* isolates harbored ARG against beta-lactams (*bla*_CTX-M-15_ and *bla*_OXA-1_) as well as aminoglycosides, fluoroquinolones, macrolide-lincosamide-streptogamin B, phenicols, sulfonamides, tetracycline, and trimethoprim. The *E*. *cloacae* genomes contained ARG against beta-lactams (*ampC*, *bla*_CTX-M-15,_ *bla*_OXA-1_ and *bla*_TEM-1B_), and aminoglycosides, fluoroquinolones, fosfomycin, phenicols, sulfamethoxazole, tetracycline, and trimethoprim. The *C*. *freundii* genome contained ARG against beta-lactams (*ampC* and *bla*_TEM-1B_) as well as against aminoglycosides, sulfonamides, and trimethoprim. The genomic context of the ESBL gene *bla*_CTX-M-15_ and the broad-spectrum beta-lactamase genes *bla*_OXA-1_ and *bla*_TEM-1B_ in the isolates are described further in the subsequent section.

**Table 2:** Antibiotic resistance phenotype and resistance genes in sequenced strains from Awba wetland in Ibadan, Nigeria.

Strains	MIC (μg/mL)					ARG detected by WGS analysis
	ATM	CAZ	CIP	IMP	SMX	
*Escherichia coli* CC8, CC33, CC43, CC46, CC57, CC78, CC85	48	32	>32	0.25	>1024	*aac(3)-IIa*, *aac(6’)-lb-cr*, *aph(3”)-Ib*, *aadA5*, *aph(6”)-Ib*, *bla*_CTX-M-15,_ *bla*_OXA-1_, *catB4*, *dfrA17*, *mphA*, *sul1*, *sul2*, *tet(B)*
*Enterobacter cloacae* CC14, CC80, CC81, CC90	16	16	3	0.38	>1024	*aac(3)-IIa*, *aac(6’)-lb-cr*, *aph(3”)-Ib*, *aadA1*, *aph(6”)-Ib*, *bla*_CMH-3_ (*ampC*), *bla*_CTX-M-15,_ *bla*_OXA-1_, *bla*_TEM-1B,_ *catA1*, *catB4*, *dfrA14*, *fosA*, *oqxA*, *oqxB*, *sul2*, *tetA*
*Citrobacter freundii* CC12	0.05	0.38	0.38	0.38	>1024	*aadA2*, *aph(3”)-Ib*, *aph(6”)-Ib*, *bla*_CMY-89_ (*ampC*), *bla*_TEM-1B,_ *dfrA18*, *sul1*, *sul2*

ATM: aztreonam, CAZ: ceftazidime, CIP: ciprofloxacin, IMP: imipenem, and SMX: sulfamethoxazole

*aac*: aminoglycoside acetyltransferase gene, *aadA*: aminoglycoside adenylyltransferase gene, *aph*: aminoglycoside phosphotransferase gene, *bla*: beta-lactamase gene, *cat*: fenicol transferase gene, *dfrA*: dihydrofolate reductase, *fosA*: fosfomycin resistance protein, *mphA*: macrolide inactivation gene, *oqx*: coding for multidrug efflux pump, active on fluoroquinolones, *sul*: dihydropteroate synthase gene, *tet*: tetracycline efflux transporter gene

In the *E*. *cloacae* isolates and the *C*. *freundii* strain, *ampC* and the genes *ampR*, *ampE*, *ampG*, and *ampD* involved in *ampC* induction were at conserved loci on contigs with high synteny and sharing up to 98% nucleotide sequence similarity with chromosomal regions in several fully sequenced reference genomes [e.g. *E*. *cloacae* strain SBP-8 (CP016906) and *C*. *freundii* strain CFNIH1 (NZ_CP007557)], respectively. The *ampC* sequences in the *E*. *cloacae* reference and our *E*. *cloacae* isolates were identical; while in *C*. *freundii* CC12 the gene was 99.65% similar to *bla*_CMY-89_ (NG_048886). A phylogenetic tree of representative AmpC amino acid sequences in our isolates and reference strains is shown in S1 Fig. We could not find sequence-based evidence for constitutive expression of *ampC* in our strains [70–72], indicating that the presence of this gene did not contribute to 3GC resistance in the isolates.

All the isolates carrying *bla*_CTX-M-15_ showed resistance (MIC range in parenthesis) to ATM (16–48μg/mL), CAZ (16–48μg/mL), CIP (3->32μg/mL) and SMX (>1024μg/mL). The *bla*_TEM-1B_ producing *C*. *freundii* CC12 was sensitive to all the antibiotics tested except SMX. None of the isolates showed resistance to imipenem.

### The genes *bla*_CTX-M-15_, *bla*_TEM-1B_ and *bla*_OXA-1_ were present on IncF, IncH and IncR plasmids

**Fig 1:**
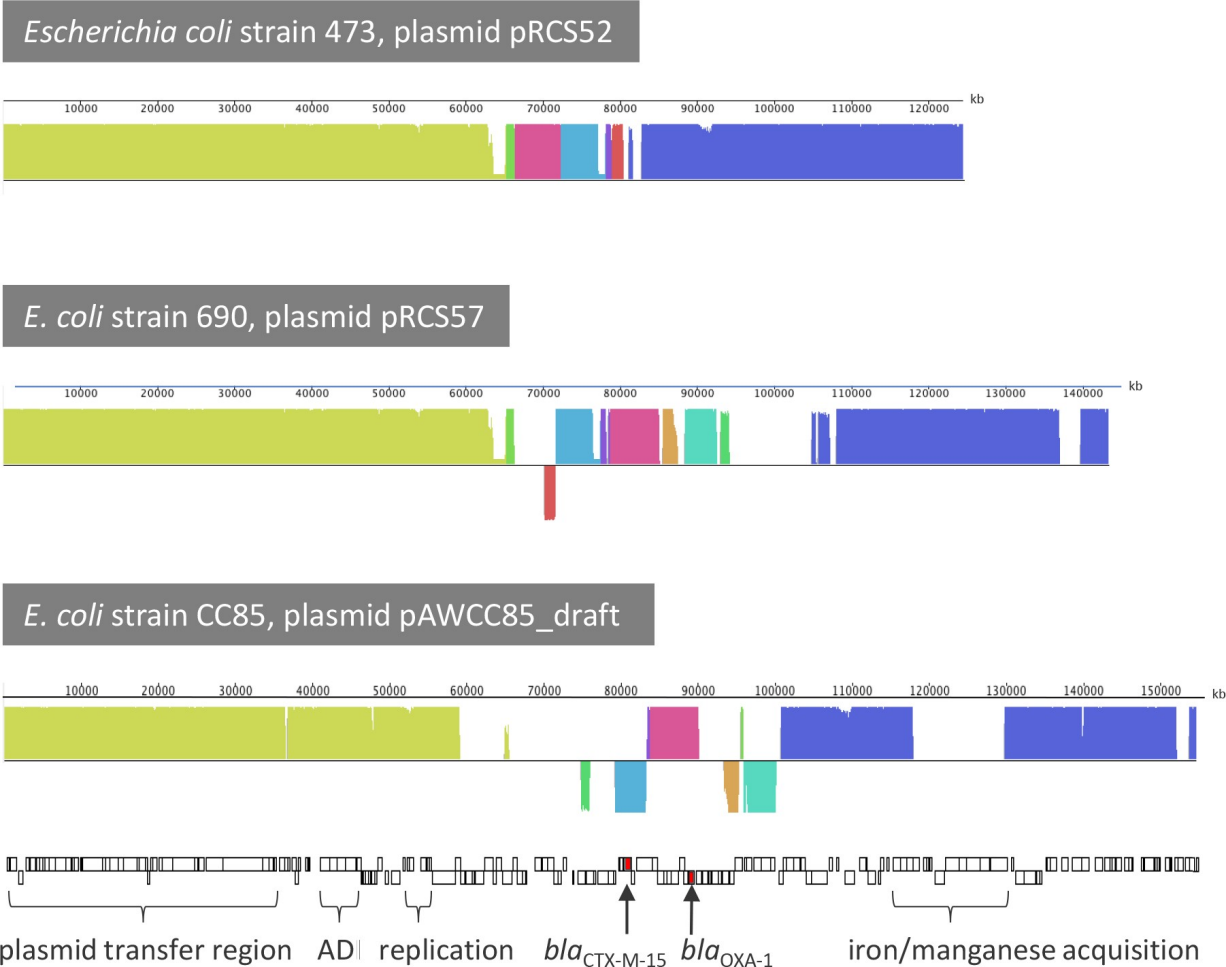
Mauve similarity plots of pAWCC85_draft and the closest homologs pCCS52 and pRCS57. The height of the Locally Collinear Blocks (LCB) shows the degree of variation between conserved regions in these plasmids. Mauve’s LCB connecting lines are not shown. Annotated genes of pAWCC85_draft are indicated as rectangles below the similarity plots. Beta-lactamase genes are shown as red rectangles; the acronym ADI stands for arginine deiminase pathway.

Draft assemblies of the various plasmids in the *E*. *coli*, *E*. *cloacae* and *C*. *freundii* isolates were generated *via* iterative MAUVE alignments with IncF, IncH, and IncR reference plasmids and mapping of the Illumina sequencing reads against the references and draft assemblies. The plasmid assemblies of the *E*. *coli* and *E*. *cloacae* isolates, respectively, were identical. Representative assemblies from the Awba isolates *E*. *coli* CC85 (pAWCC85_draft), *E*. *cloacae* CC14 (pAWCC14_draft), and *C*. *freundii* CC12 (pAWCC12_draft) were therefore used for further analysis. The pAWCC85_draft of about 155 kb in size is most similar to the IncF plasmid pRCS57 from *E*. *coli* strain 690 isolated from a urine sample collected in France [73](Fig 1). The backbone region of pAWCC85_draft encompassing genes for the conjugative apparatus, the arginine deiminase pathway (ADI pathway), metal acquisition, and plasmid maintenance together with various genes of unknown function had 99.9% nucleotide sequence identity (99% coverage) with pRCS57. Such high levels of sequence identities in plasmids from environmental isolates have rarely been found before [74]. The presence of genes coding for the ADI pathway and for metal acquisition may contribute to the virulence of the host cell and foster the epidemiological success of IncF plasmids [73,75]. The accessory region contained all 13 ARG detected in the WGS conferring resistance to beta-lactams including 3GC (*bla*_CTX-M-15_, *bla*_OXA-1_), aminoglycosides, fluoroquinolones, macrolide-lincosamide-streptogamin B, phenicol, sulfonamides, and tetracycline. The entire ARG region harbors multiple copies of genes coding for transposases, integrases, and other insertion elements, due to which the positional order of the ARG-containing contigs was uncertain and four assembly gaps remained at insertion elements encoding for a transposase domain protein, integrase core domain protein, uncharacterized insertion element, and *ISE*cp18 transposase, respectively.

There are a few differences between pAWCC85_draft and the closest known relative, pRCS57. In the latter as in many other IncF plasmids the *bla*_CTX-M-15_ gene is inserted into a target site in the *tnpA* of a *bla*_TEM-1_-Tn*2* transposon [76]. This transposon is not present in the entire WGS of the *E*. *coli* isolates from Awba. Further, the tetracycline resistance gene *tetA* is coupled to transposon Tn*1721* in pRCS57 whereas pAWCC85_draft carries a *tet(B)*-Tn*10* transposon. In pAWCC85_draft there is a class I integron with *dfrA17*, *aadA5*, and a conserved 3’ region whereas pRCS57 harbors a class I integron which is truncated after *dfrA14* by *tnpA*. Furthermore, pAWCC85_draft carries *aph (3")-lb*, *aph(6)-ld*, and *sul2*, all of which are not present in pRCS57. Next to these differences, the genes for aerobactin biosynthesis and uptake of the iron-aerobactin complex are present in pAWCC85_draft as well as other IncF plasmids used as reference (e.g. *E*. *coli* strain AR_0014 plasmid unitig_1_pilon; CP024860) but not in pRCS57.

To support the overall structure of the draft assembly and to test for transmissibility of the presumptive IncF plasmid in the *E*. *coli* isolates, conjugation assays were carried out using *A*. *aquatica* AE235 as recipient strain. Conjugation frequencies were 2 x 10^−4^. The *A*. *aquatica* transconjugants grew in LB broth and on agar plates with 50 μg/ml ceftriaxone or100 μg/ml kanamycin while growth of the parent strain AE235 was completely inhibited with 12.5 μg/ml of either antibiotic. PCR assays confirmed the presence of plasmid backbone genes (*traA*, *traN*, *traU*, *trbB*) and all ARG included in the assembly in the transconjugants.

**Fig 2:**
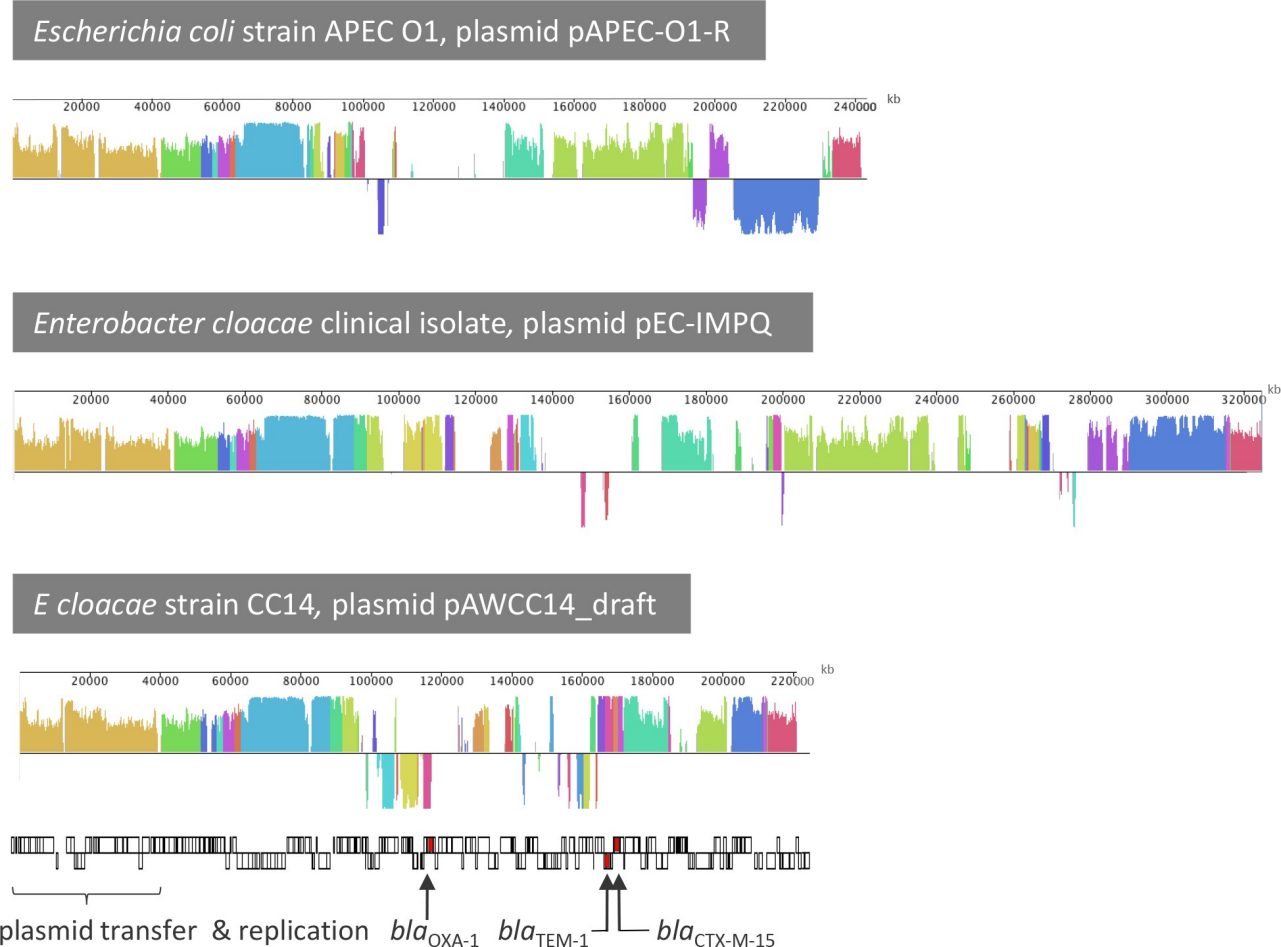
Mauve similarity plots of pAWCC14_draft and the homologs pAPEC-O1-R and pEC-IMPQ.

The IncH-type assembly pAWCC14_draft (scaffold with 15 contigs) from the *E*. *cloacae* isolates has a size of about 221 kb (Fig 2), which is within the range of sizes reported for these plasmids [16]. Organization and sequence similarity among the IncH reference plasmids and pAWCC14_draft were not as high as with the IncF plasmids. The backbone region containing the genes for conjugation and plasmid maintenance in pAWCC14_draft was most similar to pAPEC-O1-R (NC_009838) from the extraintestinal pathogenic *E*. *coli* strain APEC O1 [77]. Similar to other IncH plasmids, pAWCC14_draft contains genes for resistance against copper (*pco*), nickel/cobalt (*rcn*), and tellurite (*ter*). Close homologs to silver resistance genes (*sil*) present on some IncH plasmids [78] including pAPEC-O1-R were however found on a 293 kb contig of apparent chromosomal origin in the *E*. *cloacae* isolates. The assembly pAWCC14_draft harbored 15 out of the 19 ARG identified in the *E*. *cloacae* genomes, namely the ESBL genes *bla*_CTX-M-15_, *bla*_OXA-1_, *bla*_TEM-1B_, as well as those conferring resistance to aminoglycosides (*aadA1*, *acc(3’)-IIa*, *aph(3")-lb*, *aph(6)-ld*), fluoroquinolones (*acc(6)lb-cr*, *oqxA*, *oqxB*, *qnrB1*), phenicols (*catA1*, *catB4*), sulfonamide (*sul2*), and trimethoprim (*dfrA14*). The *bla*_CTX-M-15_ gene together with genes coding for a cupin-fold metalloprotein and a fragment of a *Tn3* family transposase were identical in the *E*. *cloaceae* and *E*. *coli* isolates as well as many plasmid sequences in the GenBank, e.g. pRCS57. Likewise, the *bla*_OXA-1_ gene together with *catB4* and *acc(6)lb-cr* were identical in the pAWCC14_draft and pAWCC85_draft, and 99% similar to the homologues in many sequenced IncF and IncH plasmids. In successful conjugation assays with *A*. *aquatica* as recipient strain, conjugation frequencies were 6 x 10^−5^. The presence of IncH backbone genes and all ARG on the contigs were confirmed by PCR.

**Fig 3:**
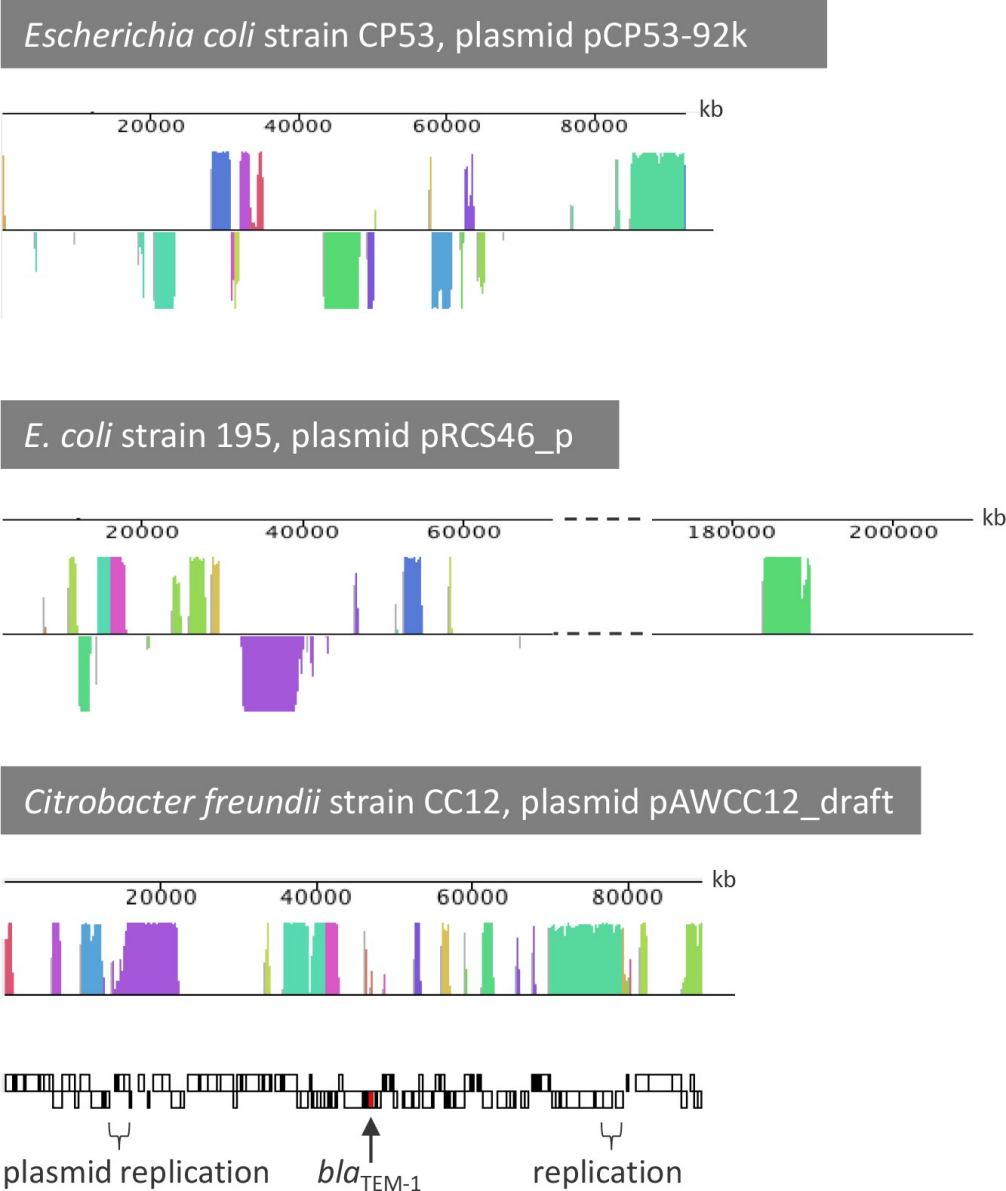
Mauve similarity plots of pAWCC12_draft and the homologs pCP53-92k and pRCS46_p. Of the latter plasmid, the non-homologous region between 70 kb and 170 kb is not shown.

The IncR-type pAWCC12_draft (16 contigs) in *C*. *freundii* CC12 is 85 kb in size (Fig 3), which is similar to the size of 9 out of the 23 reference plasmids used for generating the assembly scaffold. The size of pAWCC12_draft is a conservative estimate as many described IncR plasmids contain prophages. At least10 prophages appear to be present in the WGS of isolate CC12 but there was no sufficient evidence that any of these are part of pAWCC12_draft. The draft assembly contained genes for plasmid replication, maintenance and stability but no recognizable conjugation system could be detected. Consistent with this, conjugation assays with *A*. *aquatica* were not successful. The inability to be transferred *via* conjugation together with the absence of a discernable conjugation system was already described for IncR plasmids [79]. Based on plasmid sequence comparisons and extensive read mapping the pAWCC12_draft assembly contains the *bla*_TEM-1B_ gene and ARG conferring resistance against aminoglycosides (*aadA2*, *aph (3")-lb*, *aph(6)-ld*), sulfonamides (*sul1*, *sul2*) and trimethoprim (*dfrA19*). The *bla*_TEM-1B_ gene and an adjacent *Tn3* family transposase gene (*tnpR*) were identical in pAWCC12_draft and pAWCC14_draft, and several plasmid sequences deposited in the GenBank such as pEC-IMPQ [80].

### Abundances of marker genes in the wetlands

ESBL genes *bla*_CTX-M-1_, *bla*_TEM_, *bla*_*SHV*_, and *ampC* were assayed as a marker for clinically relevant ARG contamination in the wetland sample. Only *bla*_CTX-M_ and *ampC* were detected in samples from Awba, samples from the other sampling sites tested negative for all assayed genes. The abundances of *bla*_CTX-M-1_ and *ampC* were then enumerated in the AW samples using qPCR.

*bla*_CTX-M-1_ could only be reliably quantified in the January 2015 samples with an absolute abundance of 2x10^4^±9x10^3^ copy numbers per gram of wetland sediment and normalized abundance relative to bacterial 16S rRNA gene copy numbers of 1x10^-4^. Measured abundances in the other Awba samples were not considered due to low amplification efficiencies (around 70%). Isolation of *bla*_CTX-M_ containing strains from samples taken in November 2014, December 2014 and January 2015 together with amplification in standard PCR demonstrated that microbes harboring this gene were present at Awba at several time points. The absolute and relative abundances of *bla*_CTX-M-1_ measured at the site were similar to that reported for the sediment of Lake Brêt, Switzerland [81], an urban river in Kinshasa, Democratic Republic of Congo [82], and in waste water treatments plants and urban wetlands in the United States [83], but slightly lower than that measured in a river receiving hospital effluents in Tamil Nadu, India [84]. In all of those studies primers targeting Group 1 *bla*_CTX-M_ were used.

The *ampC* gene was detected in the AW sediment samples collected November 2014 and January 2015, where all the sequenced *ampC*-bearing bacteria were isolated from, with absolute abundance (copy number per gram of sediment) of 3.8x10^5^±2.9x10^4^ and 1.3x10^5^±8.8x10^3^, and normalized abundance relative to the bacterial 16S rRNA gene of 2.1x10^-2^ and 1.5x10^-4^ respectively. The absolute abundance was similar to what was observed in a river estuary [85] in Shanghai, China. The normalized abundance (3.9x10^-6^ to 5x10^-6^) was 2 to 4 orders of magnitude higher in the Nigerian sediments than in the sediments from the Chinese estuary and 2–6 orders of magnitude higher than in sediments from the Saudi Arabian red sea coast where normalized abundances of 7.5x10^−8^ to 9.1x10^−6^ were measured [86].

Occurrences of *bla*_CTX-M-1_ and *ampC* correlated with the qPCR-based enumeration of *E*. *coli* abundance using the *uidA* gene as marker, which was detected in the AW samples at all time points but not at the other sites. Copy numbers of *uidA* per gram of sediment at AW ranged from 4.3x10^4^ to 2.6x10^5^, while the relative abundance normalized against bacterial 16S rRNA gene copy numbers ranged from 3.8x10^-5^ to 2.8x10^-4^.

## Conclusions

In this study, we found multi-drug resistant and potentially pathogenic *E*. *coli* belonging to the globally distributed ST10 complex harboring *bla*_CTX-M-15_ on a self-transmissible IncF plasmid in a polluted urban wetland (Awba) in Ibadan, southwestern Nigeria. The wetland also harbored multi-drug resistant *E*. *cloacae* with *bla*_CTX-M-15_ on a self-transmissible IncH plasmid, and *C*. *freundii* with *bla*_TEM-1B_ on an IncR plasmid. Previously, carbapeneme-resistant *Pseudomonas* spp. harboring *bla*_VIM-5_ on novel class I integrons were isolated from the same wetland [10], further indicating a substantial level of contamination of this wetland with clinically relevant ARG. The wetland drains into a reservoir that is used as source of domestic water supply and may potentially contribute to the spread of AMR in the human population. Given the insufficient status of local sanitation infrastructure these findings on AMR prevalence are not overly surprising, but it is hoped that these results will be a catalyst for infrastructure improvements to prevent dissemination of ARG into the aquatic ecosystem in this region.

**Fig 4:**
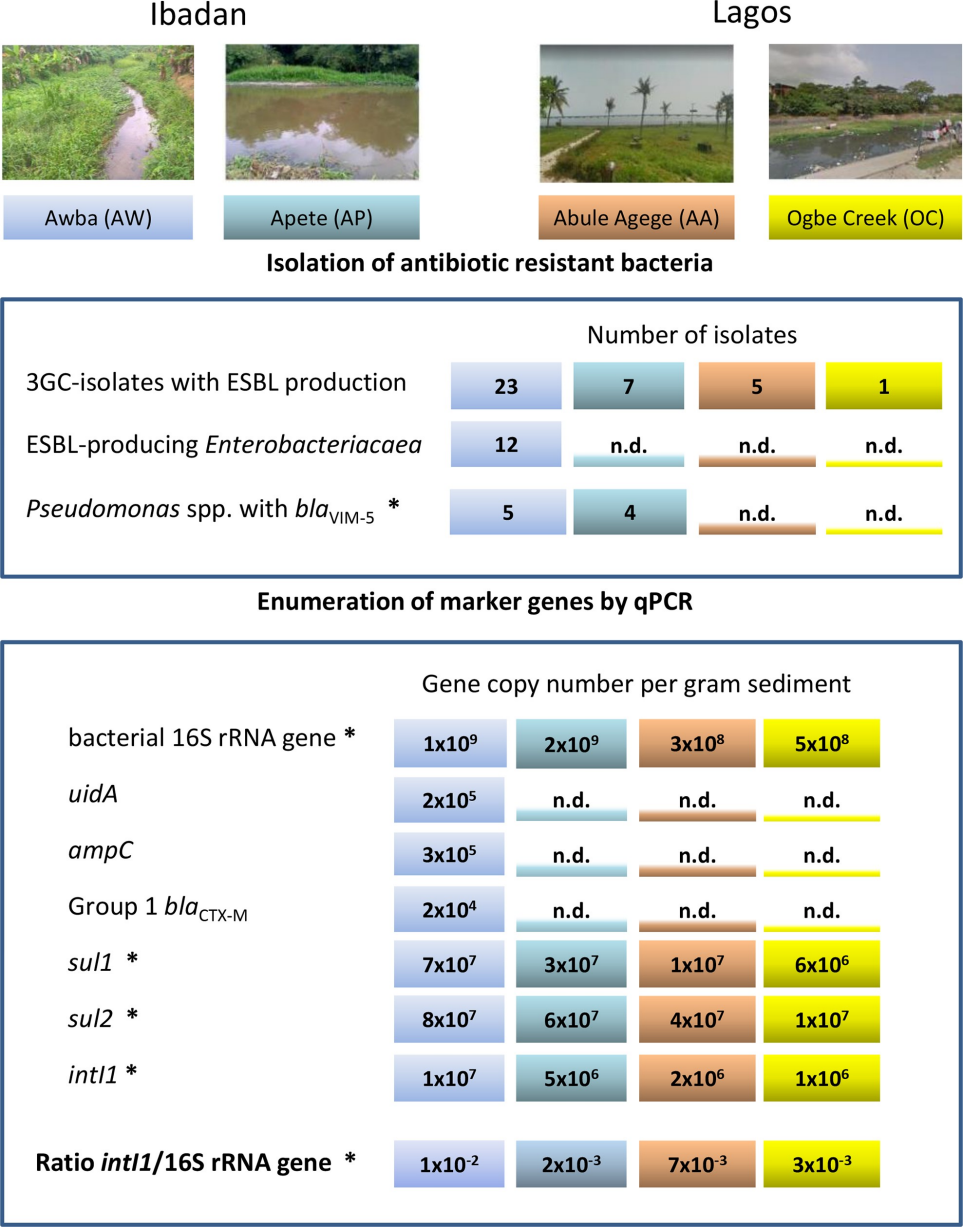
Results of AMR profiling for four urban wetlands in southwestern Nigeria. Data marked with an asterisk are from [10] and [12]. Standard deviations for qPCR-based enumerations of marker genes are provided in the main narrative of this and the two previous publications, and n.d. stands for “not detected”.

We further note that the copy number of *intI1* to bacterial 16S rRNA gene abundance correlated with the observed level of AMR prevalence in the four investigated urban wetlands [12] (Fig 4). The *intI1* gene is among several indicators suggested for monitoring anthropogenic AMR pollution in the environment [4,87]. Although we analyzed only a small number of samples taken over a short time frame, results from this and our previous studies on the four Nigerian wetlands suggest that determining the *intI1*/16S rRNA gene copy number ratio may be sufficient for generating maps of AMR distribution in the environment, including the presumptive identification of local hotspots. Such maps could guide were to carryout detailed analyses involving cultivation or metagenomics as described by Hendricksen *et al*., [8] to provide a picture of the AMR profile in the human community. In LIC and LMIC, detailed knowledge on environmental prevalence of AMR might stand as proxy for a more-costly national surveillance program which involves extensive sampling in the clinical setting. It could also indicate which antibiotics are no more useful for treatment due to high levels of resistance as well as identify newly emerging resistance determinants in the region. The availability of prevalence maps could also be helpful to direct resources for sanitation infrastructure improvements to where they would be most cost-effective for combating the spread of AMR.

## Supporting information

S1 Table Reference plasmids used for generating the draft assemblies pAWCC85_draft (IncF-type), pAWCC14_draft (IncH-type) and pAWCC12_draft (IncR-type). (DOCX)

S2 Table Primers used in this study. (DOCX)

S1 Fig Phylogenetic dendrogram of representative AmpC sequences deposited in GenBank including those from the present study. (DOCX)
