# Supplementary material for: A survey of extended-spectrum beta-lactamase-producing Enterobacteriaceae in urban wetlands in southwestern Nigeria as a step towards generating prevalence maps of antimicrobial resistance
Source: PLoS One. 2020 Mar 4;15(3):e0229451. doi: 10.1371/journal.pone.0229451 (PMC7055906; doi:10.1371/journal.pone.0229451)
Supplement: S1 Table — (DOCX) [file pone.0229451.s001.docx]

**Supporting Information**

**A survey of extended-spectrum beta-lactamase-producing *Enterobacteriaceae* in urban wetlands in southwestern Nigeria as a step towards generating prevalence maps of antimicrobial resistance**

Olawale Olufemi Adelowo^1,2*^, Odion Osebhahiemen Ikhimiukor^1,2^, Camila Knecht^1,3^, John Vollmers^4^, Mudit Bhatia^1^, Anne-Kirstin Kaster^4^ and Jochen A. Müller^1*^

^1^Department of Environmental Biotechnology, Helmholtz Centre for Environmental Research - UFZ, Leipzig, Germany

^2^Environmental Microbiology and Biotechnology Laboratory, Department of Microbiology, University of Ibadan, Ibadan, Nigeria

^3^Otto-von-Guericke-Universität Magdeburg - Institute of Apparatus and Environmental Technology, Magdeburg, Germany

^4^Institute for Biological Interfaces (IBG5), Karlsruhe Institute of Technology, Eggenstein-Leopoldshafen, Germany

*Corresponding authors:

OOA: e-mail: [onomewaleadelowo@yahoo.co.uk](mailto:onomewaleadelowo@yahoo.co.uk), [oo.adelowo@ui.edu.ng](mailto:oo.adelowo@ui.edu.ng)

JAM: email: [jochen.mueller@ufz.de](mailto:jochen.mueller@ufz.de)

**S1 Table:** Reference plasmids used for generating the draft assemblies pAWCC85_draft (IncF-type), pAWCC14_draft (IncH-type) and pAWCC12_draft (IncR-type)

| **Plasmid name** | **size (bp)** | **Plasmid host** | **Isolation source** | **Geographic location** | **Accession number** |
| --- | --- | --- | --- | --- | --- |
| **IncF-type reference plasmids** | | | | | |
| pRCS52 | 124,392 | *Escherichia coli* strain 473 | *Homo sapiens* | France | [LO017736](https://www.ncbi.nlm.nih.gov/nuccore/LO017736) |
| pRCS57 | 143,225 | *E. coli* strain 690 | *Homo sapiens* | France | [LO017738](https://www.ncbi.nlm.nih.gov/nuccore/1036082041) |
| pM309-NDM5 | 136,947 | *E. coli* M309 | *Homo sapiens* | Yangon, Myanmar | [AP018833](https://www.ncbi.nlm.nih.gov/nuccore/AP018833) |
| pCA14 | 155,456 | *E. coli* strain CA14 | *Homo sapiens* | USA | [CP009231](https://www.ncbi.nlm.nih.gov/nuccore/CP009231) |
| pKp_Goe_414-6 | 57,266 | *Klebsiella pneumoniae* isolate Kp_Goe_154414 | *Homo sapiens* | Lower Saxony, Germany | [CP018343](https://www.ncbi.nlm.nih.gov/nuccore/CP018343) |
| pEC517_1 | 118,495 | *E. coli* strain Ecol_517 | *Homo sapiens* | Rio de Janeiro, Brazil | [CP018964](https://www.ncbi.nlm.nih.gov/nuccore/CP018964) |
| pECAZ146_1 | 150,994 | *E. coli* strain Ecol_AZ146 | *Homo sapiens* | Pisa, Italy | [CP018990](https://www.ncbi.nlm.nih.gov/nuccore/CP018990) |
| pECAZ155_KPC | 272,202 | *E. coli* strain Ecol_AZ155 | *Homo sapiens* | Beijing, China | [CP019001](https://www.ncbi.nlm.nih.gov/nuccore/CP019001) |
| pECAZ162_KPC | 142,829 | *E. coli* strain Ecol_AZ162 | *Homo sapiens* | Boston, USA | [CP019014](https://www.ncbi.nlm.nih.gov/nuccore/CP019014) |
| p1493-5 | 127,772 | *E. coli* strain CRE1493 | *Homo sapiens* | Shenzhen, China | [CP019076](https://www.ncbi.nlm.nih.gov/nuccore/CP019076) |
| pDA33133-157 | 156,518 | *E. coli* strain DA33133 | *Homo sapiens* | Sweden | [CP029575](https://www.ncbi.nlm.nih.gov/nuccore/CP029575) |
| plasmid unitig_1_pilon | 172,588 | *E. coli* strain AR_0014 | missing information | missing information | [CP024860](https://www.ncbi.nlm.nih.gov/nuccore/CP024860) |
| **IncH-type reference plasmids** | | | | | |
| pAPEC-O1-R | 241,387 | *E. coli* APEC O1 | *Meleagris sp.*  (turkey) | USA | [NC_009838](https://www.ncbi.nlm.nih.gov/nuccore/NC_009838) |
| pEC-IMPQ | 324,503 | *Enterobacter cloacae* | *Homo sapiens* | Taiwan | [EU855788](https://www.ncbi.nlm.nih.gov/nuccore/EU855788) |
| pENVA | 253,984 | *K. pneumoniae* Kp15 | *Felissilvestriscatus*  (domestic cat) | France | [HG918041](https://www.ncbi.nlm.nih.gov/nuccore/HG918041) |
| pNDM-MAR | 267, 242 | *K. pneumoniae* | *Homo sapiens* | Morocco | [JN420336](https://www.ncbi.nlm.nih.gov/nuccore/JN420336) |
| **IncR-type reference plasmids** | | | | | |
| pCP53-92k | 92,168 | *E. coli* strain CP53 | pig feces | Sichuan, China | [CP033095](https://www.ncbi.nlm.nih.gov/nuccore/CP033095) |
| plasmid RCS46_p | 216,620 | *E. coli* strain 195 | missing information | missing information | [LT985249](https://www.ncbi.nlm.nih.gov/nuccore/LT985249) |
| pNCYU-26-73-2 | 128,115 | *E. coli* strain NCYU-26-73 | *Susscrofa domesticus* | Taiwan | [CP042617](https://www.ncbi.nlm.nih.gov/nuccore/CP042617) |
| pHYEC-mcr1 | 97,559 | *E. coli* strain HYEC7 | pig feces | Guandong, China | [KX518745](https://www.ncbi.nlm.nih.gov/nuccore/KX518745) |
| pR15_MCR-1 | 109,908 | *E. coli* strain R15 | wastewater | China | [MK256965](https://www.ncbi.nlm.nih.gov/nuccore/MK256965) |
| pCHL5009T-88k | 88,388 | *E. coli* strain CHL5009T | *Homo sapiens* | Christchurch, New Zealand | [CP032939](https://www.ncbi.nlm.nih.gov/nuccore/CP032939) |
| *E. coli* strain O177:H21 plasmid unnamed3 | 126,046 | *E. coli* strain O177:H21 | *Homo sapiens* | Leiden, Netherlands | [CP016549](https://www.ncbi.nlm.nih.gov/nuccore/CP016549) |
| plasmid A | 94,395 | *E. coli* strain H8 | river water | missing information | [CP010173](https://www.ncbi.nlm.nih.gov/nuccore/CP010173) |
| pHNSD133T1 | 129,713 | *E. coli* strain SDX5C133 | chicken | China | [MG196293](https://www.ncbi.nlm.nih.gov/nuccore/MG196293) |
| *E. coli* strain A1_181 plasmid p_unnamed2 | 97,582 | *E. coli* strain A1_181 | *Larus sp.*  (gull) | Anchorage, Alaska | [CP040069](https://www.ncbi.nlm.nih.gov/nuccore/CP040069) |
| pEFER | 55,150 | *Escherichia fergusonii* ATCC 35469 | human feces | Missouri, USA | [CU928144](https://www.ncbi.nlm.nih.gov/nuccore/CU928144) |
| pSA20030575 | 94,179 | *Salmonella enterica* strain SA20030575 | missing information | missing information | [CP030183](https://www.ncbi.nlm.nih.gov/nuccore/CP030183) |
| pSa76-CIP | 104,666 | *Salmonella sp.* strain Sa76 | food product | China | [MG874044](https://www.ncbi.nlm.nih.gov/nuccore/MG874044) |
| plasmid unnamed1 | 125,137 | *Citrobacter portucalensis* strain Effluent_1 | wastewater | Cholsey, United Kingdom | [CP039328](https://www.ncbi.nlm.nih.gov/nuccore/CP039328) |
| pCROD1 | 54,449 | *Citrobacter rodentium* ICC168 | laboratory mouse | USA | [FN543503](https://www.ncbi.nlm.nih.gov/nuccore/FN543503) |
| pKN-0c4e | 61,178 | *K. pneumoniae* strain KPNIH49 | wastewater/sludge | USA | [CP026182](https://www.ncbi.nlm.nih.gov/nuccore/CP026182) |
| pK66-45-1 | 338,512 | *K. pneumoniae* strain K66-45 | *Homo sapiens* | Norway | [CP020902](https://www.ncbi.nlm.nih.gov/nuccore/CP020902) |
| pKP1780 | 49,770 | *K. pneumoniae*  strain KP-1780 | *Homo sapiens* | Greece | [JX424614](https://www.ncbi.nlm.nih.gov/nuccore/JX424614) |
| pKPC-LK30 | 86,518 | *K. pneumoniae* | *Homo sapiens* | Taiwan | [KC405622](https://www.ncbi.nlm.nih.gov/nuccore/KC405622) |
| pKPS30 | 61,228 | *K. pneumoniae* | *Homo sapiens* | Paris, France | [KF793937](https://www.ncbi.nlm.nih.gov/nuccore/KF793937) |
| pKPS77 | 45,867 | *K. pneumoniae* strain KPS77 | *Homo sapiens* | Paris, France | [KF954150](https://www.ncbi.nlm.nih.gov/nuccore/KF954150) |
| pSCKLB684-mcr | 93,227 | *K. pneumoniae* strain SCKLB684 | missing information | missing information | [MH781719](https://www.ncbi.nlm.nih.gov/nuccore/MH781719) |
| *K. pneumoniae* strain KLB08 plasmid sequence | 96,564 | *K. pneumoniae* strain KLB08 | missing information | missing information | [MK112268](https://www.ncbi.nlm.nih.gov/nuccore/MK112268) |
