# Supplementary material for: A survey of extended-spectrum beta-lactamase-producing Enterobacteriaceae in urban wetlands in southwestern Nigeria as a step towards generating prevalence maps of antimicrobial resistance
Source: PLoS One. 2020 Mar 4;15(3):e0229451. doi: 10.1371/journal.pone.0229451 (PMC7055906; doi:10.1371/journal.pone.0229451)
Supplement: S2 Table — (DOCX) [file pone.0229451.s002.docx]

**Supporting Information**

**A survey of extended-spectrum beta-lactamase-producing *Enterobacteriaceae* in urban wetlands in southwestern Nigeria as a step towards generating prevalence maps of antimicrobial resistance**

Olawale Olufemi Adelowo^1,2*^, Odion Osebhahiemen Ikhimiukor^1,2^, Camila Knecht^1,3^, John Vollmers^4^, Mudit Bhatia^1^, Anne-Kirstin Kaster^4^ and Jochen A. Müller^1*^

^1^Department of Environmental Biotechnology, Helmholtz Centre for Environmental Research - UFZ, Leipzig, Germany

^2^Environmental Microbiology and Biotechnology Laboratory, Department of Microbiology, University of Ibadan, Ibadan, Nigeria

^3^Otto-von-Guericke-Universität Magdeburg - Institute of Apparatus and Environmental Technology, Magdeburg, Germany

^4^Institute for Biological Interfaces (IBG5), Karlsruhe Institute of Technology, Eggenstein-Leopoldshafen, Germany

*Corresponding authors:

OOA: e-mail: [onomewaleadelowo@yahoo.co.uk](mailto:onomewaleadelowo@yahoo.co.uk), [oo.adelowo@ui.edu.ng](mailto:oo.adelowo@ui.edu.ng)

JAM: email: [jochen.mueller@ufz.de](mailto:jochen.mueller@ufz.de)

**Table S2.** Primers used in this study

| **Primer** | **Primer sequence 5’ – 3’** | **Melting**  **temperature [°C]** | **Product size**  **[bp]** | **Reference** |
| --- | --- | --- | --- | --- |
| **targeting IncF backbone** | | | | |
| traA-F | GGACCTGATGGCAAGCGGTA | 56 | 187 | this study |
| traA-R | AACGACGGCCATACCCACAG |  |  |  |
| traX-F | TTAACCGGCTGTGGGGATGG | 58 | 389 | this study |
| traX-R | TGCACACGACACCAGACCAA |  |  |  |
| trbB-F | CTCTGGCATCCGCAGGGTAT | 57 | 163 | this study |
| trbB-R | GCCCCTGCATAAACAGCACC |  |  |  |
| **targeting IncH backbone** | | | | |
| traU-F | TGACAGCAACTGAAGCCGGA | 56 | 295 | this study |
| traU-R | CCATCGCATCCGGCACAAAA |  |  |  |
| traN-F | ACCGCACCCTCGTAAATGCT | 58 | 285 | this study |
| traN-R | ACATGTCTCCGTTTCCGCCT |  |  |  |
| tnpR-F | CGCCCTGAGCGTCAAATTCC | 57 | 159 | this study |
| tnpR-R | CCGATAAGGCATCCGGCAGT |  |  |  |
| **targeting ARG in plasmids** | | | | |
| aadA5-F | TTTTCAAGCAGCTGTCCCAC | 62 | 250 | this study |
| aadA5-R | GCGACAACAGTTAGCTCCAG |  |  |  |
| aac3-IIa-F | CGGAAGGCAATAACGGAG | 62 | 740 | [1] |
| aac3-IIa-R | TCGAACAGGTAGCACTGAG |  |  |  |
| aac(6')-Ib-cr-F | TTGCGATGCTCTATGAGTGGCTA | 61 | 482 | [2] |
| aac(6')-Ib-cr-R | CTCGAATGCCTGGCGTGTTT |  |  |  |
| ampC_F | CCTCTTGCTCCACATTTGCT |  | 189 | [3] |
| ampC_R | ACAACGTTTGCTGTGTGACG |  |  |  |
| aph(6’’)-Id-F | CGTTTCGCAACCTGTTCTC | 52 | 509 | this study |
| aph(6’’)-Id-R | CGCAGTTCATCAGCAATGTC |  |  |  |
| aph(3")-Ib-F | TGCTAACGCCGAAGAGAAC | 52 | 548 | this study |
| aph(3")-Ib-R | CAATCGCAGATAGAAGGCAAG |  |  |  |
| tetRB_F | GATCCCTGAAAGCAAACGGC | 60 | 967 | this study |
| tetRB_R | AGGCCGAATAAGAAGGCTGG |  |  |  |
| **Screening of ARGs in non-ESBL producing isolates** | | | | |
| TEM-F | TCGCCGCATACACTATTCTCAGAATGAC | 58 | 422 | [4] |
| TEM-R | CAGCAATAAACCAGCCAGCCGGAAG |  |  |  |
| SHV-F | TGTATTATCTC(C/T)CTGTTAGCC(A/G)CCCTG | 58 | 739 | [4] |
| SHV-R | GCTCTGCTTTGTTATTCGGGCCAAGC |  |  |  |
| CTX-M-F | ATGTGCAGYACCAGTAARGTKATGGC | 58 | 590 | [4] |
| CTX-M-R | GGTRAARTARGTSACCAGAAYCAGCGG |  |  |  |
| VEB-F | GATGGTGTTTGGTCGCATATCGCAAC | 58 | 391 | [4] |
| VEB-R | CATCGCTGTTGGGGTTGCCCAATTTT |  |  |  |
| GES-F | CTGGCAGGGATCGCTCACTC | 58 | 604 | [4] |
| GES-R | GGTTTCCGATCAGCCACCTCTCA |  |  |  |
| PER-F | CAGTGTGGGGGCCTGACGAT | 58 | 731 | [4] |
| PER-R | CTGAGCAACCTGCGCAATRATAGCTT |  |  |  |
| CMY-1-F | GCTGCTCAAGGAGCACAGGATCCCG | 60 | 522 | [5] |
| CMY-1-R | GGCACATTGACATAGGTGTGGTGCATG |  |  |  |
| CMY-2-F | ACTGGCCAGAACTGACAGGCAAA | 60 | 466 | [5] |
| CMY-2-R | GTTTTCTCCTGAACGTGGCTGGC |  |  |  |
| ACC-F | TCCAGCCGCTGATGCAGAAGAAT | 60 | 365 | [5] |
| ACC-R | CCAYGCTTTTAGATAAGCCATCAGCTG |  |  |  |
| ACT-F | TCGGTAAAGCCGATGTTGCGG | 60 | 302 | [5] |
| ACT-R | CTTCCACTGCGGCTGCCAGTT |  |  |  |
| DHA-F | CTTTCACAGGTGTGCTGGGTGCG | 60 | 403 | [5] |
| DHA-R | CCG TAC GCA TAC TGG CTT TGC GC |  |  |  |
| FOX-F | CAT GGG GTA TCA GGG AGA TGC C | 60 | 218 | [5] |
| FOX-R | GCCGCTGCTCGCCCATCG |  |  |  |
| OXA-1-F | CAACGGATTAACAGAAGCATGGCTCG | 60 | 198 | [5] |
| OXA-1-R | GCTGTRAATCCTGCACCAGTTTTCCC |  |  |  |
| OXA-2-F | GACCAAGATTTGCGATCAGCAATGCG | 60 | 256 | [5] |
| OXA-2-R | CYTTGACCAAGCGCTGATGTTCYACC |  |  |  |
| OXA-10-F | CGCCAGAGAAGTTGGCGAAGTAAG | 60 | 138 | [5] |
| OXA-10-R | GAAACTCCACTTGATTAACTGCGG |  |  |  |
| OXA-23-F | CCTGATCGGATTGGAGAACCAG | 60 | 516 | [5] |
| OXA-23-R | GATGCCGGCATTTCTGACCG |  |  |  |
| OXA-24-F | GGTCGATAATTTTTGGTTAGTTGGCCC | 60 | 237 | [5] |
| OXA-24-R | CCATTAGCTTGCTCCACCCAACCAG |  |  |  |
| OXA-48-F | CCAAGCATTTTTACCCGCATCKACC | 60 | 389 | [5] |
| OXA-48-R | GYTTGACCATACGCTGRCTGCG |  |  |  |
| OXA-51-F | GACCGAGTATGTACCTGCTTCGACC | 60 | 497 | [5] |
| OXA-51-R | GAGGCTGAACAACCCATCCAGTTAACC |  |  |  |
| OXA-58-F | GTGCTGAGCATAGTATGAGTCGAGC | 60 | 630 | [5] |
| OXA-58-R | GGTCTACAGCCATTCCCCAGCC |  |  |  |
| **targeting ARGs in sediment samples** | | | | |
| CTX-M-1 Forward | SCSATGTGCAGYACCAGTAA | 58 | 543 | [3] |
| CTX-M-1 Reverse | CCGCRATATGRTTGGTGGTG |  |  |  |
| CTX-M-1 PCR UP | ACGTTAACACCGCCATTCC | 58 | 356 | [6] |
| CTX-M-1 PCR LP | TCGGTGACGATTTTAGCCGA |  |  |  |
| CTX-M-1-qPCR UP | ACCAACGATATCGCGGTGAT | 60 | 100 | [6] |
| CTX-M-1-qPCR LP | ACATCGCGACGGCTTTCT |  |  |  |
| SHV-F | TGTATTATCTC(C/T)CTGTTAGCC(A/G)CCCTG | 58 | 739 | [4] |
| SHV-R | GCTCTGCTTTGTTATTCGGGCCAAGC |  |  |  |
| TEM-1 Forward | CATTTTCGTGTCGCCCTTAT | 58 | 167 | [6] |
| TEM-1 Reverse | GGGCGAAAACTCTCAAGGAT |  |  |  |
| TEM PCR UP | CTACCCAGAAACGTGGTG | 58 | 569 | [6] |
| TEM PCR LP | ATCCGCCTCCATCCAGTCTA |  |  |  |
| **targeting phylogenetic marker (*uidA*)** | | | | |
| Eco-F | CTGCTGCTGTCGGCTTTA | 60 | 205 | [7] |
| Eco-R | CCTTGCGGACGGGTAT |  |  |  |

**References**

1. Soleimani N, Aganj M, Ali L, Shokoohizadeh L, Sakinc T. Frequency distribution of genes encoding aminoglycoside modifying enzymes in uropathogenic *E. coli* isolated from Iranian hospital. BMC Research Notes. 2014 Dec;7(1):842.
2. Park CH, Robicsek A, Jacoby GA, Sahm D, Hooper DC. Prevalence in the United States of *aac (6′)-Ib-cr* encoding a ciprofloxacin-modifying enzyme. Antimicrobial Agents and Chemotherapy. 2006 Nov 1;50(11):3953-5.
3. Yang Y, Zhang T, Zhang XX, Liang DW, Zhang M, Gao DW, Zhu HG, Huang QG, Fang HH. Quantification and characterization of β-lactam resistance genes in 15 sewage treatment plants from East Asia and North America. Applied Microbiology and Biotechnology. 2012 Sep 1;95(5):1351-8.
4. Trung NT, Hien TT, Huyen TT, Quyen DT, Binh MT, Hoan PQ, Meyer CG, Velavan TP. Simple multiplex PCR assays to detect common pathogens and associated genes encoding for acquired extended spectrum beta-lactamases (ESBL) or carbapenemases from surgical site specimens in Vietnam. Annals of Clinical Microbiology and Antimicrobials. 2015 Dec;14(1):23.
5. Voets GM, Fluit AC, Scharringa J, Stuart JC, Leverstein-van Hall MA. A set of multiplex PCRs for genotypic detection of extended-spectrum β-lactamases, carbapenemases, plasmid-mediated AmpC β-lactamases and OXA β-lactamases. International Journal of Antimicrobial Agents. 2011 Apr 1;37(4):356-9.
6. Colomer-Lluch M, Jofre J, Muniesa M. Antibiotic resistance genes in the bacteriophage DNA fraction of environmental samples. PLOS ONE. 2011 Mar 3;6(3):e17549.
7. Kaushik R, Balasubramanian R. Assessment of bacterial pathogens in fresh rainwater and airborne particulate matter using Real-Time PCR. Atmospheric Environment. 2012 Jan 1;46:131-9.
