# Supplementary material for: A survey of extended-spectrum beta-lactamase-producing Enterobacteriaceae in urban wetlands in southwestern Nigeria as a step towards generating prevalence maps of antimicrobial resistance
Source: PLoS One. 2020 Mar 4;15(3):e0229451. doi: 10.1371/journal.pone.0229451 (PMC7055906; doi:10.1371/journal.pone.0229451)
Supplement: S1 Fig — (DOCX) [file pone.0229451.s003.docx]

**Supporting Information**

**A survey of extended-spectrum beta-lactamase-producing *Enterobacteriaceae* in urban wetlands in southwestern Nigeria as a step towards generating prevalence maps of antimicrobial resistance**

Olawale Olufemi Adelowo^1,2*^, Odion Osebhahiemen Ikhimiukor^1,2^, Camila Knecht^1,3^, John Vollmers^4^, Mudit Bhatia^1^, Anne-Kirstin Kaster^4^ and Jochen A. Müller^1*^

^1^Department of Environmental Biotechnology, Helmholtz Centre for Environmental Research - UFZ, Leipzig, Germany

^2^Environmental Microbiology and Biotechnology Laboratory, Department of Microbiology, University of Ibadan, Ibadan, Nigeria

^3^Otto-von-Guericke-Universität Magdeburg - Institute of Apparatus and Environmental Technology, Magdeburg, Germany

^4^Institute for Biological Interfaces (IBG5), Karlsruhe Institute of Technology, Eggenstein-Leopoldshafen, Germany

*Corresponding authors:

OOA: e-mail: [onomewaleadelowo@yahoo.co.uk](mailto:onomewaleadelowo@yahoo.co.uk), [oo.adelowo@ui.edu.ng](mailto:oo.adelowo@ui.edu.ng)

JAM: email: [jochen.mueller@ufz.de](mailto:jochen.mueller@ufz.de)

**
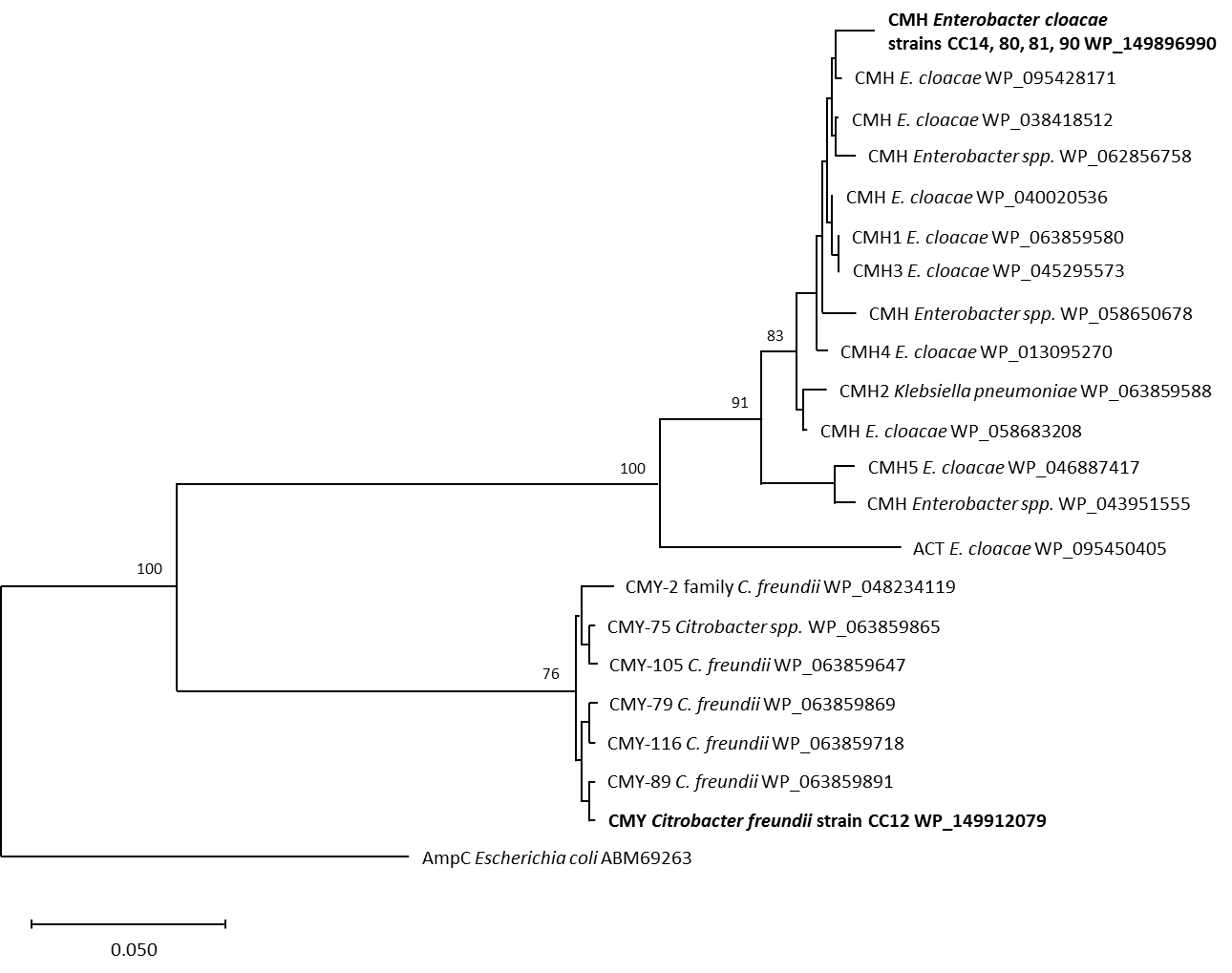
**

**Fig S1.Phylogenetic dendrogram of representative AmpC sequences deposited in GenBank including those from the present study.**
